# Coinfection of *Chlamydia psittaci* and *Enterococcus faecalis* exacerbated respiratory distress in patients: from isolation to mouse model

**DOI:** 10.3389/fcimb.2025.1662902

**Published:** 2025-10-10

**Authors:** Xuedi Zhang, Yihui Wang, Yuhan Wang, Yuehui Cui, Huimin Wang, Zongyang Huang, Lin Luo, Linlin Tang, Jianlin Chen, Cheng He

**Affiliations:** ^1^ National Key Laboratory of Veterinary Public Healthy Security, College of Veterinary Medicine, China Agricultural University, Beijing, China; ^2^ Department of Critical Care Medicine, The Second People’s Hospital, Changde, Hunan, China; ^3^ Reproductive Medicine Centre, Department of Obstetrics and Gynecology, The Second Xiangya Hospital, Central South University, Changsha, Hunan, China

**Keywords:** *Chlamydia psittaci*, *Enterococcus faecalis*, respiratory distress, coinfection, patient

## Abstract

**Background:**

This study aimed to isolate and identify *Chlamydia psittaci* (*C. psittaci*) and *Enterococcus faecalis* (*E. faecalis*) from a patient suspected of community-acquired pneumonia.

**Methods:**

The samples from the patient (lung lavage and throat swab) and his son (throat swab) were tested to determine antibodies against COVID-19 and *C. psittaci*-specific IgG. Afterward, 40 female mice were inoculated intranasally with coinfection of *C. psittaci* and *E. faecalis* and primary infection of *C. psittaci* followed by *E. faecalis*. Meanwhile, eight mice with *C. psittaci* and *E. faecalis* infection alone served as the control group. Clinical signs, lung lesions, and pathogen loads were monitored.

**Results:**

Positive *C. psittaci* genomics were detected in both the patient’s lung lavage and his son’s swabs, while *C. psittaci* and *E. faecalis* were isolated and identified from the patient’s lung lavage samples. Moreover, positive *C. psittaci*-specific IgG and negative COVID-19 antibodies were determined. The patient recovered after 10-day doxycycline treatment. Mice showed weight loss, breathing difficulties, and diffuse alveolar damage after inoculation with *C. psittaci* followed by *E. faecalis*.

**Conclusion:**

Our experiment demonstrated that coinfection, particularly sequential infection with *C. psittaci* followed by *E. faecalis*, can duplicate severe respiratory distress and typical pathological lesions.

## 1 Introduction

Since December 8, 2019, several cases of unknown respiratory disease have been reported in Wuhan, Hubei Province, China. Subsequently, Changde, Beijing, Shanghai, Japan, and Korea reported severe febrile respiratory illness that spread to household members and healthcare workers (Ciotti et al., 2019; Islam et al., 2022). Clinically, it was difficult to differentiate between novel coronavirus and *Chlamydia psittaci* infection, with similar symptoms including high fever, sore throat, and dry cough (Cianfarani, 2019; Jin et al., 2021). Moreover, *C. psittaci* infection is similar to other types of community-acquired pneumonia, including *Chlamydia pneumoniae*, *Legionella pneumophila*, *Mycoplasma pneumoniae*, and human metapneumovirus (Qin et al., 2022; Liu et al., 2023). In 2020, four medical workers were diagnosed with *C. psittaci* pneumonia during routine COVID-19 screening in the Second Xiangya Hospital, Hunan, China (Lei et al., 2021).

A 50-year-old farmer who was a patient at the time also experienced severe breathing difficulties with a high fever. The patient had an initial medical history of heart disease and diabetes for up to 7 years. Following his sickness, he was admitted to the Second People’s Hospital of Changde, Hunan, China, on December 13, 2019, and was suspected of having avian influenza, as he was raising 50 yellow-feather broilers in his backyard. After hospital admission, he received antivirals for 5 days, and his high fever persisted for an additional week with respiratory distress, including headache, asthma, and chest pains. Five days later, qPCR revealed that he was negative for avian influenza virus and COVID-19. However, positive *C. psittaci* was detected in lung lavage samples using qPCR assay and whole-genome sequencing. Based on the diagnosis, the sick farmer and his son received 10 mg/kg of doxycycline via injection for 10 days and recovered in the hospital. The patient, his son, and his spouse had positive *C. psittaci*-specific IgG antibodies after hospitalization. Meanwhile, *C. psittaci* and *Enterococcus faecalis* were isolated and identified from the patient’s lung lavage samples.

*C. psittaci* is an important zoonotic agent with a wide host spectrum, and it causes systemic infection. Its unique biphasic developmental cycle and persistent state assist in the survival and immune escape of host cells. Acute pneumonia (psittacosis) caused by *C. psittaci* infection is frequently misdiagnosed and poses significant challenges in treatment (Wang et al., 2024). Infected hosts (both animal and human) show a diversity of clinical signs, from asymptomatic disease for a large portion of infected organisms to multiple organ failure, sepsis, and death. In 2018, 13 workers were reported to be infected in a U.S. poultry slaughterhouse, indicating a high risk of zoonotic transmission of psittacosis (Shaw et al., 2019). In recent years, human psittacosis has increased gradually due to the commercial application of whole-genome sequencing and increasing patient survival after therapy (Wu et al., 2021). In December 2020, six employees from a duck meat processing plant and two unemployed people were diagnosed with community-acquired pneumonia and tested positive for *C. psittaci* using nested PCR and qPCR, indicating human-to-human transmission of *C. psittaci* in China and an emergent risk (Zhao et al., 2021; Zhang et al., 2022).

However, the pathogenesis between prolonged severe respiratory distress and coinfection of *C. psittaci* and *E. faecalis* is unknown. The purpose of this study was to illustrate the potential contribution of a combination of *C. psittaci* and *E. faecalis* in driving critical respiratory distress.

## 2 Materials and methods

### 2.1 Temperature curve and clinical samples

A patient with community-acquired pneumonia was admitted to a designated hospital in Changde, Hunan, China, on December 13, 2019, and an alert was triggered twice due to two waves of high fever on December 20 and December 25–30, 2019. One week later, while caring for his father, the patient’s 30-year-old son also had a high fever. After treatment with doxycycline for 11 days, both his high fever and breathing difficulty were under control (**Supplementary Figure S1**). Finally, he was discharged from the local hospital before the COVID-19 outbreak in Wuhan, approximately 260 km away from his hometown. During his hospital admission, samples were collected with his family’s consent (**Supplementary Table S1**). Moreover, swabs, sera, and an alveolar wash were collected from his relatives and close contacts (**Supplementary Table S2**). Before collecting any clinical samples, signed informed consent was sought from possible participants, including family members, the attending physician, and the nurses. Also, the study was approved by the institutional review board (IRB) of China Agricultural University. The collected throat swabs were kept in sucrose-phosphate-glutamate (SPG) solution at 4°C, while the sera were stored at −20°C for further testing.

### 2.2 COVID-19 and *Chlamydia* tests

All serum samples were detected using a commercial antibody kit (Lizhu Biotech Co., Ltd., Shenzhen, China), and RNA samples were analyzed in accordance with the qPCR protocol recommended by the WHO (Corman et al., 2020) using COVID-19 primers (**Supplementary Table S3**).

Genomic sequences were amplified for *Chlamydia* tests using specific primers (**Table 1**). The *Chlamydia* genus was determined using the following: 2× super mix 12.5 µL, 10 µM *Chlamydia* forward primer 1.5 µL, 10 µM reverse primer 1.5 µL, and 0.5–1 µg DNA. The PCR conditions were conducted as follows: 95°C, 5 min, denaturing at 95°C for 30 s, annealing at 56°C for 30 s, and extending at 72°C for 30 s. The cycle was repeated 30 times, and the final reaction was incubated at 72°C for 10 min. Whole-genome sequencing was performed by a commercial institute (Beijing Genomics Institution, BGI, Beijing, China). For *C. psittaci* detection, the *ompA* gene was determined using qPCR (Applied Biosystems™ 7500, Thermo Fisher, Beijing, China) as described previously (Fang et al., 2021). The qPCR reagents included 5 µL SYBR Reaction Mix (2×) (TransGen Biotech, Beijing, China), 1 µL forward primer (2.5 μM), 1 µL reverse primer (2.5 μM), 1 µL template DNA, and 2 µL ddH_2_O. qPCR was performed as follows: 50°C for 10 min, 95°C for 5 min, 95°C for 10 s, 60°C for 30 s, 45 cycles of 95°C for 1 min, 55°C for 1 min, and 55°C–95°C (0.5°C increment) for 10 s. The *ompA* gene of *C. psittaci*, approximately 1,209 bp, was amplified as described previously (Fang et al., 2021). It was subjected to electrophoresis in a 1% (w/v) agarose gel. The PCR product was sequenced by a commercial company (Tsingke Biotechnology Co., Ltd., Beijing, China).

**Table 1:** Primers of sequences used for the detection of *Chlamydia* species.

S/N	Primers	Sequence (5′–3′)	Specificity and amplicon length
1	Ch23S-F	CTGAAACCAGTAGCTTATAAGCGGT (25 nt)	Chlamydiaceae (111 bp)
	Ch23S-R	ACCTCGCCGTTTAACTTAACTCC (23 nt)	
	Ch23S-p	FAM-CTCATCATGCAAAAGGCACGCCG-TAM	
2	Cp.ps.OMP1-F	CACTATGTGGGAAGGTGCTTCA	*Chlamydia psittaci* (76 bp)
	Cp.ps.OMP1-R	CTGCGCGGATGCTAATGG	
	Cp.ps.OMP1-S	FAM-CGCTACTTGGTGTGAC-BHQ1 (MGB-Sonde)	
3	F1-incA-Cpsi	GCCATCATGCTTGTTTCGTTT	*C. psittaci* (74 bp)
	R1-incA-Cpsi	CGGCGTGCCACTTGAGA	
	S-Cpsi-incA-NM	FAM-TCATTGTCATTATGGTGATTCAGGA-MGBNFQ	
4	CpaOMP1-F	GCAACTGACACTAAGTCGGCTACA	*Chlamydia abortus* (82 bp)
	CpaOMP1-R	ACAAGCATGTTCAATCGATAAGAGA	
	CpaOMP1-S	FAM-TAAATACCACGAATGGCAAGTTGGTTTAGCG-TAM	
5	CppecOMP1-F	CCATGTGATCCTTGCGCTACT	*Chlamydia pecorum* (76 bp)
	CppecOMP1-R	TGTCGAAAACATAATCTCCGTAAAAT	
	CppecOMP1-S	FAM-TGCGACGCGATTAGCTTACGCGTAG-TAM	
6	Csuis23S-F	CCTGCCGAACTGAAACATCTTA	*Chlamydia suis* (118 bp)
	Csuis23S-R	CCCTACAACCCCTCGCTTCT	
	Csuis23S-S	FAM-CGAGCGAAAGGGGAAGAGCCTAAACC-TAM	
7	enoA_08-1274_F15	CAATGGCCTACAATTCCAAGAGT (23 nt)	*Chlamydia gallinacea* (72 bp)
	enoA_08-1274_R87	CATGCGTACAGCTTCCGTAAAC (22 nt)	
	enoA_08-1274_P	CY5-ATTCGCCCTACGGGAGCCCCTT-BHQ2	

The throat swabs collected from the patient and his relatives were mixed in 900 μL phosphate buffered saline (PBS) containing 2,000 IU/mL gentamicin and 2,000 IU/mL streptomycin (Solarbio Science & Technology Co., Ltd., Beijing, China). After incubation for 30 min at 37°C and centrifugation at 500 × *g* for 5 min at 4°C, the supernatant (0.4 mL) was collected and injected into 7-day-old specific pathogen free (SPF) embryonated chicken eggs at 0.2 mL per egg. Afterward, the embryonated chicken eggs were incubated at 37°C for 1 week, and the embryonated chicken eggs were monitored twice per day. The second passage was carried out to observe the pathogenicity using the yolk membranes (Zhang et al., 2009). The typical inclusion bodies were monitored using the IMAGEN™ *Chlamydia* kit (Thermo Scientific, Beijing, China), and specific species of *C. psittaci* was verified using the aforementioned qPCR. For antibody detection, *C. psittaci*-specific human antibodies were detected using SeroFIA™ *C. psittaci* immunofluorescence detection assay (Savyon Diagnostics Ltd., Ashdod, Israel) following the manufacturer’s instructions (Ling et al., 2015).

### 2.3 Identification and propagation of *E. faecalis*

DNA samples were extracted from the positive *E. faecalis* isolates using the DNeasy Tissue Kit (Qiagen, Hilden, Germany) following the manufacturer’s instructions. The following specific primers were used in the study. The forward primer was 5′-GTACAGTTGCTTCAGGACGTATC-3′, and the reverse primer was 5′-ACGTTCGATTTCATCACGTTG-3′. A 197-bp fragment of the *tuf* gene was amplified and subjected to electrophoresis in a 1% (w/v) agarose gel. The PCR procedure comprised an initial incubation for 4 min at 95°C, 35 cycles for 60 s each at 95°C, annealing for 60 s at 59°C, and extension for 60 s at 72°C, with a final extension for 5 min at 72°C (Pan et al., 2012). The PCR product was sequenced, and then NCBI blast was used.

All throat swabs were grown on standard I nutrient agar (Merck, Darmstadt, Germany) with 5% sheep blood and incubated at 37°C for 24 h. The positive colonies were transferred into the Baird–Parker medium (Qingdao Hope Bio-Technology Co., Ltd., Shandong, China). Afterward, the typical colonies were identified using Gram staining and biochemical assays (Sigma-Aldrich, Beijing, China). The biochemical tests assayed for glucose, fructose, sucrose, maltose, lactose, galactose, mannose, mannitol, sorbitol, arabinose, inulin, urease, lysine decarboxylase, nitrate, gelatinase, motility, indole, 0.1% methylene blue milk, and growth on MacConkey agar in bouillon medium at pH 9.6, sodium hippurate medium, and 6.5% NaCl bouillon medium. Bacterial colony forming unit (CFU) was quantified on nutrient agar containing 5% sheep blood (Pan et al., 2012).

### 2.4 Mice inoculated with *C. psittaci* and *E. faecalis*

The animal test was designed in accordance with guidelines issued by the Institutional Animal Care and Use Committee (IACUC) and followed humane protocols to minimize animal pain. It was approved by the Ethics Review Committee of China Agricultural University (approval code: IACUC20191222). Forty C57BL/6 mice weighing 20–22 g were randomly assigned to five groups with eight animals per group (**Supplementary Table S4**) and maintained in negative isolators in biosafety level 2 facilities. In the current study, Group 1 mice were infected intranasally with 0.1 mL of 1.0 × 10^5^ inclusion forming unit (IFU) of *C. psittaci* isolate alone. Group 2 mice were inoculated intranasally with 0.1 mL of 1.0 × 10^7^ CFU of *E. faecalis* isolate alone. Group 3 mice received intranasally 1.0 × 10^7^ CFU of *E. faecalis* and 0.1 mL of 1.0 × 10^5^ IFU of *C. psittaci* at the same time. Group 4 mice were first inoculated intranasally with 1.0 × 10^5^ IFU of *C. psittaci* isolate and then inoculated with 0.1 mL of 1.0 × 10^7^ CFU of *E. faecalis* through the same route after 3 days. This infection model was used to identify whether primary *C. psittaci* infection would aggravate *E. faecalis*-mediated respiratory distress. Group 5 mice received sterile 0.1 mL of PBS via the same route, serving as the healthy control. Mouse body weights were monitored weekly until the end of the experiment. After 14 days, the mice were anesthetized by intraperitoneal injection into the right lower quadrant of the abdomen with 2 mg ketamine and 0.2 mg xylazine (Jianglei Biotech Co., Ltd., Shanghai, China).

### 2.5 Evaluation of lung pathological lesions

Lungs were harvested from each group of euthanized mice, examined for pathological lesions, and scored. Lung lesions were determined according to a previous description (Li et al., 2022). Briefly, for lungs, the grades were as follows: grade 0, lines normal; grade 1, 30% of the lung surface area showed hemorrhage, degeneration, or necrosis; grade 2, 60% of the lung surface area showed hemorrhage, degeneration, or necrosis; and grade 3, the entire lung surface showed hemorrhage, degeneration, or necrosis. For lung sections, the grades were as follows: grade 0, none; grade 1, slight edema of the alveolar walls; grade 2, moderate edematous thickening of alveolar walls with occasional alveoli containing coagulated edema fluid; and grade 3, extensive occurrence of alveolar and interstitial edema.

### 2.6 Pathogen loads

The mouse lungs were aseptically removed on day 14. Briefly, sterile lung tissues of eight mice from each group were taken and then minced, and 50 mg of lung tissues from each mouse and supernatant lung homogenates were obtained. The homogenates were stored at 4°C for 40 min and then centrifuged at 2,000 rpm/min for 5 min. The supernatant was used to determine the concentration of *C. psittaci* by qPCR (Fang et al., 2021). For *E. faecalis* determination, the samples from the lungs were inoculated on standard I nutrient agar (Merck, Germany) with 5% sheep blood and incubated at 37°C for 24–48 h. Finally, the number of bacterial colonies was measured.

### 2.7 Statistical analysis

Statistical significance was analyzed using one-way ANOVA with the least significant difference (LSD) *post-hoc* test. Data were expressed as the mean ± standard deviation. All data were calculated and analyzed using SPSS v26.0 (SPSS Inc., Chicago, IL, USA). Graphs were generated using the GraphPad Prism 9 software (GraphPad Software, San Diego, CA, USA). Statistically significant differences were judged as *p* < 0.05.

## 3 Results

### 3.1 Detection of *C. psittaci* and specific antibodies from the patient and his close contacts

**Figure 1:**
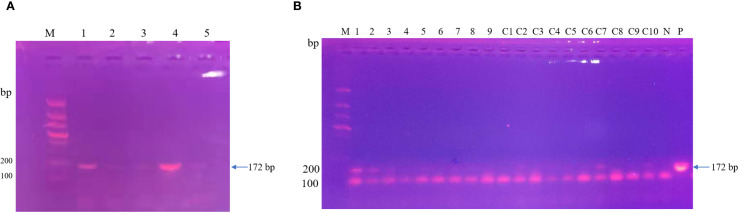
Detection of *Chlamydia psittaci* from patient’s samples and his close contacts using 23S rDNA PCR. **(A)** Positive *C*. *psittaci* was detected in the patient’s alveolar lavage (M, marker; 1, alveolar lavage; 2, throat swabs; 3, tracheal mucosa; 4, *C*. *psittaci* 6BC control; 5, negative control). **(B)** Negative samples of patient’s close contacts (M, marker; 1–7, patient’s relatives; 8, doctor; 9, nurse; C1–C10, chicken samples; N, negative control; P, positive control).

**Figure 2:**
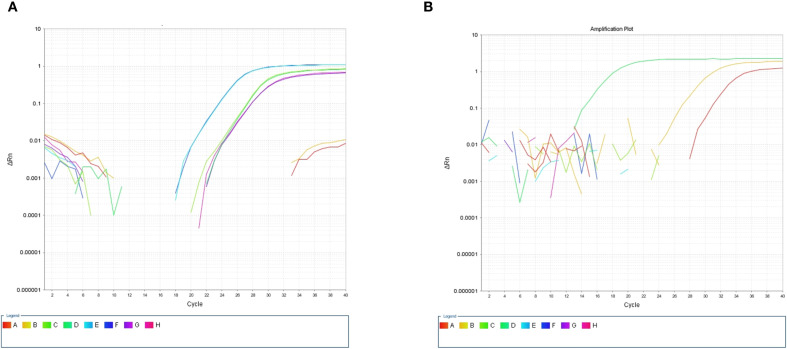
Detection of *Chlamydia psittaci* using qPCR assay. **(A)** Positive *C*. *psittaci* was detected in patient’s alveolar lavage and throat swab, while negative reaction was detected in tracheal mucosa. A, B: Tracheal mucosa, C, D: throat swabs, E, F: alveolar lavage, and G, H: *C*. *psittaci*. **(B)** Positive reaction was identified in his son’s throat swabs (D), and negative reaction was identified in his close contact’s samples: tracheal mucosa (A–C, E–H).

**Figure 3:**
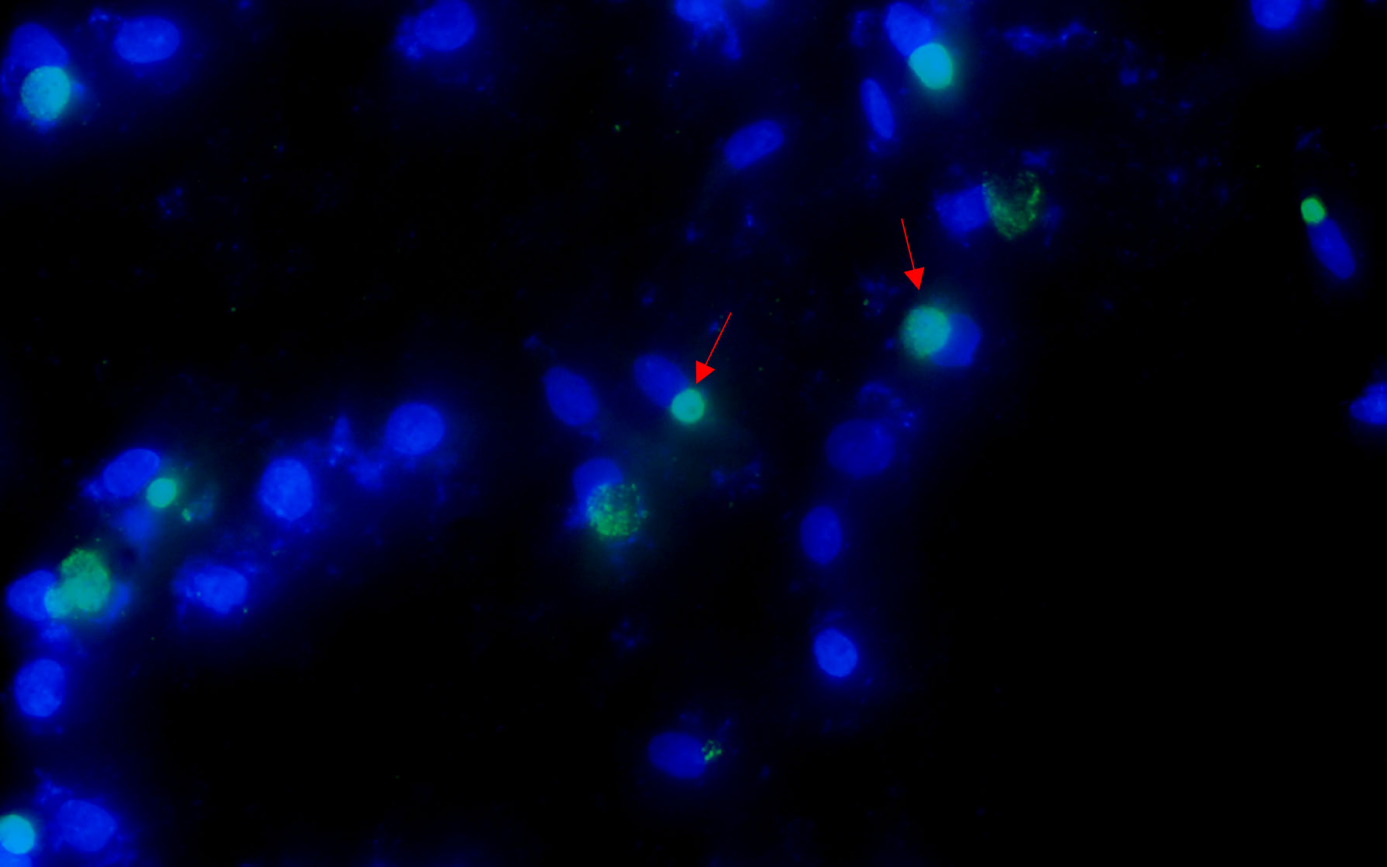
Positive *Chlamydia psittaci* strain was isolated from patient’s throat swab samples using immunofluorescence staining. Blue, 4',6-diamidino-2-phenylindole (DAPI)-stained host cells. Green, fluorescein isothiocyanate (FITC)-labeled lipopolysaccharide (LPS) antibody against *Chlamydia*.

After the patient’s hospital admission, his chest X-ray revealed bilateral pneumonia and ground-glass opacity, and he was prescribed oxygen therapy and doxycycline. Afterward, lesions with ground-glass opacity were reduced, but pulmonary consolidation was still observed in the lungs (**Supplementary Figure S2**). Based on 23S rDNA amplification, a positive 172-bp band of *C. psittaci* was detected in the patient’s alveolar wash, while his tracheal mucosa and throat swabs were negative (**Figure 1A**). Other family members, the nurses, the attending physician, and the house chickens were negative for *C. psittaci* (**Figure 1B**). Afterward, qPCR results showed that the patient’s alveolar wash was positive for *C. psittaci*. Subsequently, positive *C. psittaci* was identified in the patient’s alveolar wash and throat swab, and his sons’ throat swab. However, a negative reaction was found in his tracheal mucosa and other close contacts’ samples (**Figure 2**). The *ompA* gene sequence of the *C. psittaci* strain was determined and submitted to GenBank (accession number: OR616243). After inoculation into SPF embryonated eggs, typical intracellular inclusions were observed using immunofluorescence staining (**Figure 3**).

**Figure 4:**
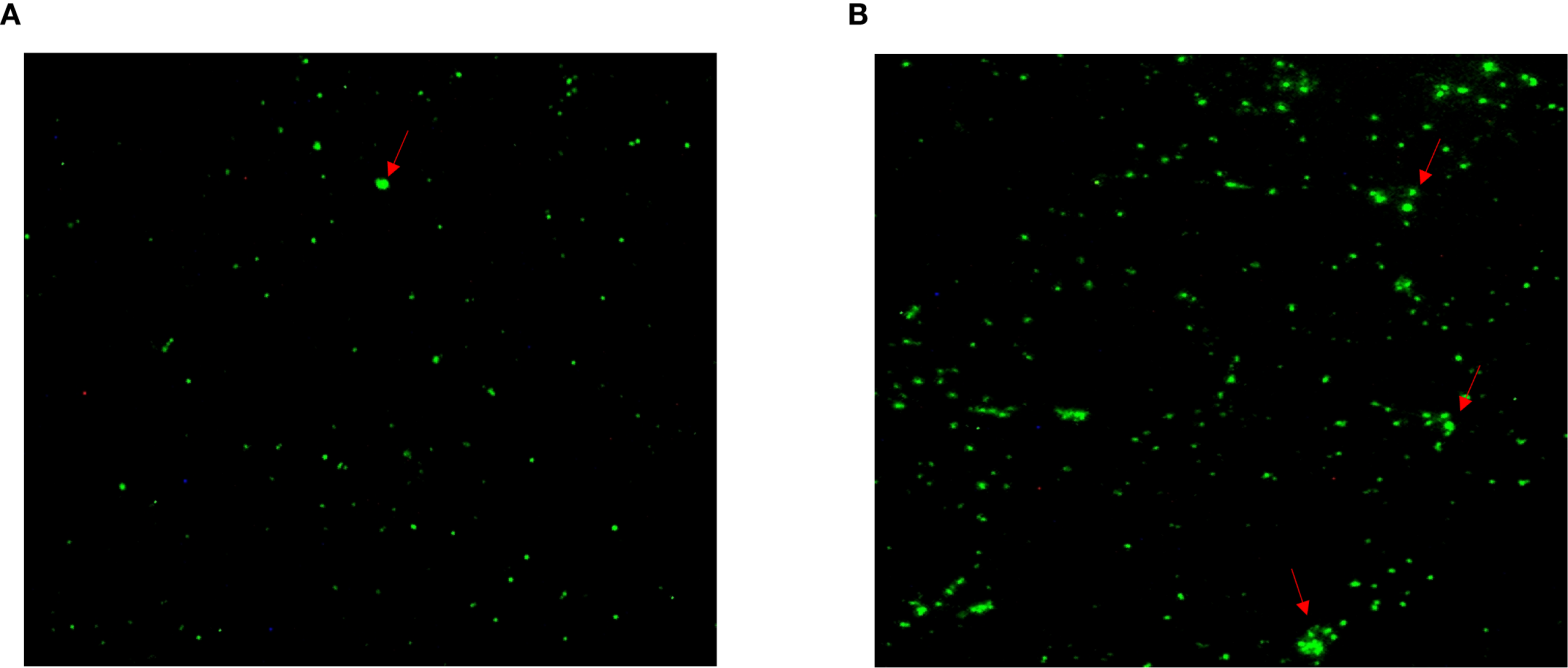
Specific antibodies against *Chlamydia psittaci* during patient’s hospital admission. **(A)** Positive antibody to *C*. *psittaci* was observed on December 25, 2019. **(B)** Highly intensive *C*. *psittaci*-specific antibodies were observed in a time-course manner on December 27, 2019.

For *C. psittaci*-specific antibodies, IgG antibody titers were arranged from 1:64 dilution on December 25 and increased to 1:128 dilution on December 27, 2019, in a time-course manner (**Figure 4**). Compared to the patient’s sera, IgG titers were from 1:32 to 1:64 to 1:128, and the serum samples from other close contacts were negative in the test. Meanwhile, antibodies against COVID-19 were negative (**Table 2**). Serum samples from the patient were also tested for antibodies against common respiratory pathogens, including influenza A/B virus, respiratory syncytial virus, *C. pneumoniae*, and *M. pneumoniae*. All results were negative.

**Table 2:** Detection of *Chlamydia psittaci*-specific IgG and COVID-19 antibodies in the sera of the patient and his close contacts.

Code	Patient and his relatives	Gender	Age	*C. psittaci* antibody	COVID-19 antibody
1	Duan (patient)	Male	66	1:32; 1:64; 1:128 positive	Negative
2	Spouse	Female	63	Negative	Negative
3	Son	Male	35	1:32; 1:64; 1:128 positive	Negative
4	Older granddaughter	Female	9	Negative	Negative
5	Little granddaughter	Female	6	Negative	Negative
6	Daughter-in-law	Female	35	Negative	Negative
7	Nurse	Female	27	Negative	Negative
8	Doctor	Male	29	Negative	Negative

### 3.2 Isolation and characterization of *E. faecalis*

**Figure 5:**
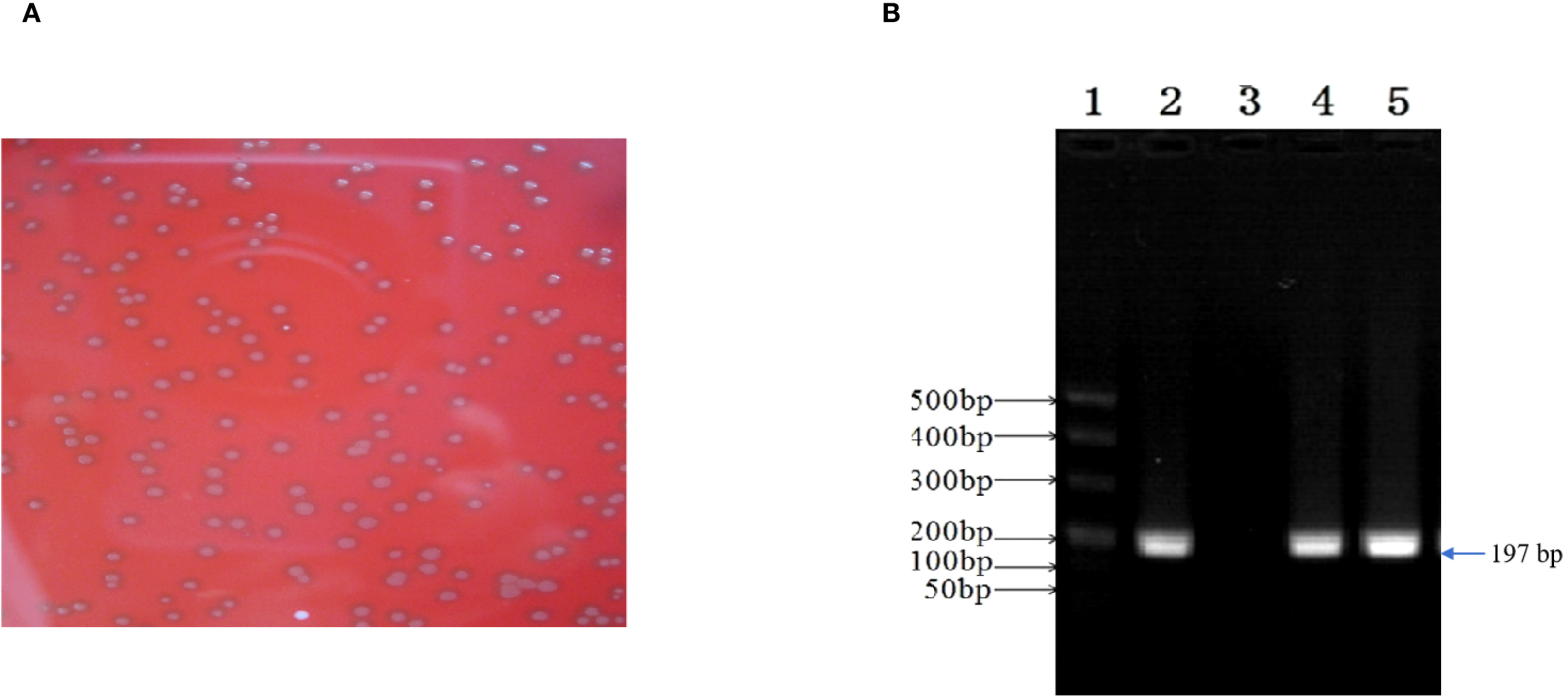
*Enterococcus faecalis* was isolated and identified from patient’s alveolar lavage and throat swabs. **(A)** Typical colonies of *E. faecalis* grew on Columbia Blood Agar Base Medium. **(B)** The isolate generated 197-bp PCR product. 1, marker; 2, positive control; 3, negative control; 4, patient’s alveolar lavage; 5, patient’s throat swab.

*E. faecalis* was successfully isolated in the alveolar lavage and throat swab using single-colony purification. Subsequently, the isolate was classified using biochemical assays and conventional PCR, and *E. faecalis* isolates were found to be Gram-positive, chain-forming, coccus-shaped organisms upon microscopic inspection (**Figure 5A**). The isolated strains were determined as *E. faecalis* using PCR. The DNA extracted from the typical colony produced the expected 197-bp PCR product of the target gene (**Figure 5B**); the sequence of the *tuf* gene was submitted to GenBank (accession number: SUB15618628), and NCBI blast was used. A sequence analysis of the *tuf* segment showed that the sequence from this isolate was 98%–100% homologous to the *E. faecalis* reference strains (GenBank # EU156939 and AY266992).

### 3.3 Pathogenicity of coinfection with *C. psittaci* and *E. faecalis*

**Figure 6:**
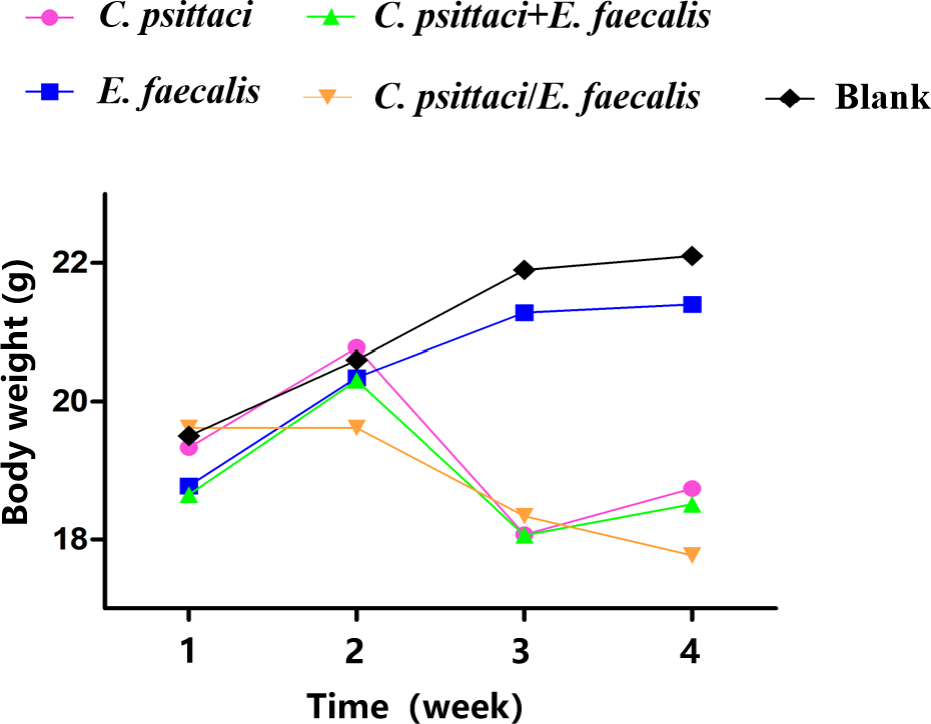
Body weight after inoculation with coinfection with *Chlamydia psittaci* or *Enterococcus faecalis* in mice. Two weeks post-infection, the body weight levels of the *C. psittaci*/*E. faecalis* group were lower than those of the other three groups (*p* < 0.01). No statistical difference was found in the *E. faecalis* alone group and the control group. The differences were analyzed using ANOVA (*p* < 0.05, *p* < 0.01).

Clinically, mice infected primarily with *C. psittaci*, followed by *E. faecalis* (*C. psittaci*/*E. faecalis*) inoculation or *C. psittaci* infection alone, showed ruffled feathers, inactivity, poor appetite, and low weight; especially severe signs were found in female mice compared to male mice. For body weights, those of the *C. psittaci*/*E. faecalis* group, *C. psittaci* alone group, and *C. psittaci*+*E. faecalis* group were lower 1 week later. More importantly, mouse body weights declined during the observation in the *C. psittaci*/*E. faecalis* group (*p* < 0.01). However, no decrease was found in the *E. faecalis* alone group and the control group (**Figure 6**).

**Figure 7:**
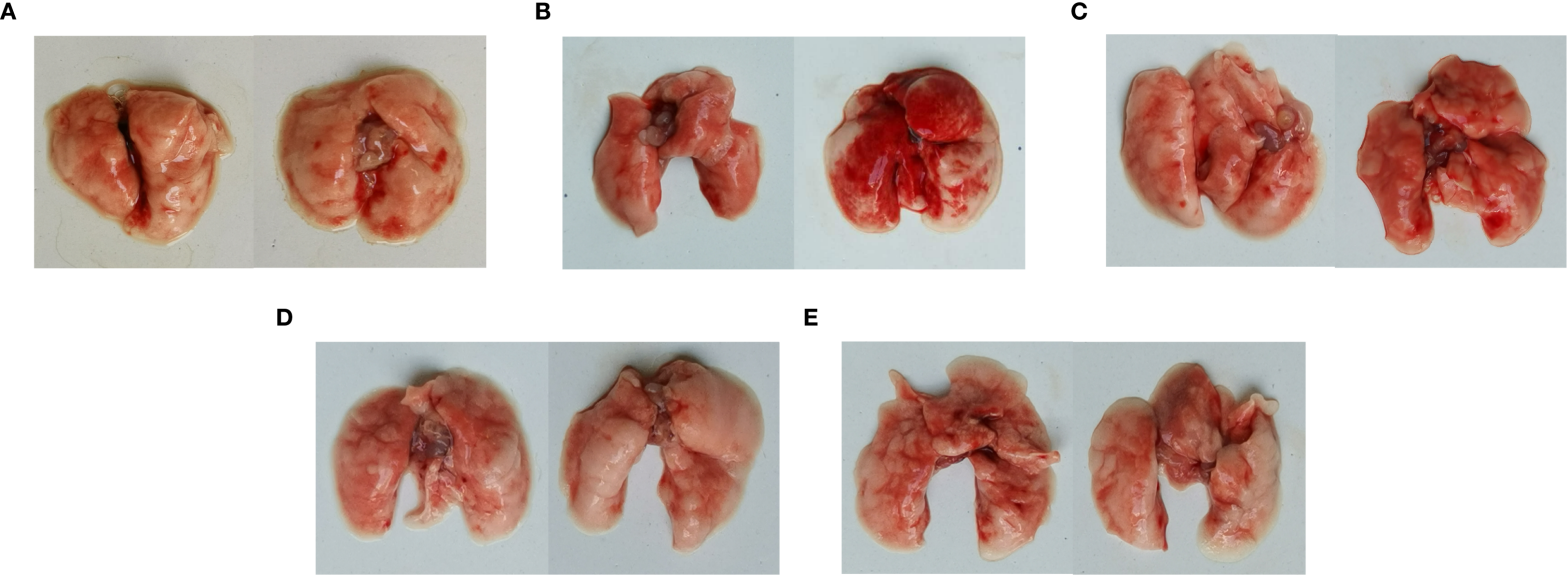
Lung lesions after coinfection with *Chlamydia psittaci* and *Enterococcus faecalis* in mice. **(A)** Control group, **(B)** *C*. *psittaci* alone group, **(C)** *E. faecalis* alone group, **(D)** *C*. *psittaci*+*E. faecalis* group, and **(E)** *C*. *psittaci*/*E. faecalis* group.

Postmortem, diffuse hemorrhagic lungs were observed in the mice with *C. psittaci* infection alone, while enlarged lung size was evident in the mice with *E. faecalis* infection (**Figure 7**). For *C. psittaci*/*E. faecalis* group and the *C. psittaci*+*E. faecalis* group, atrophy, viscous exudation, and diffuse hemorrhage characterized severe lesions, while fibrinous consolidation developed in the *C. psittaci*+*E. faecalis* group (**Figure 7D**).

**Figure 8:**
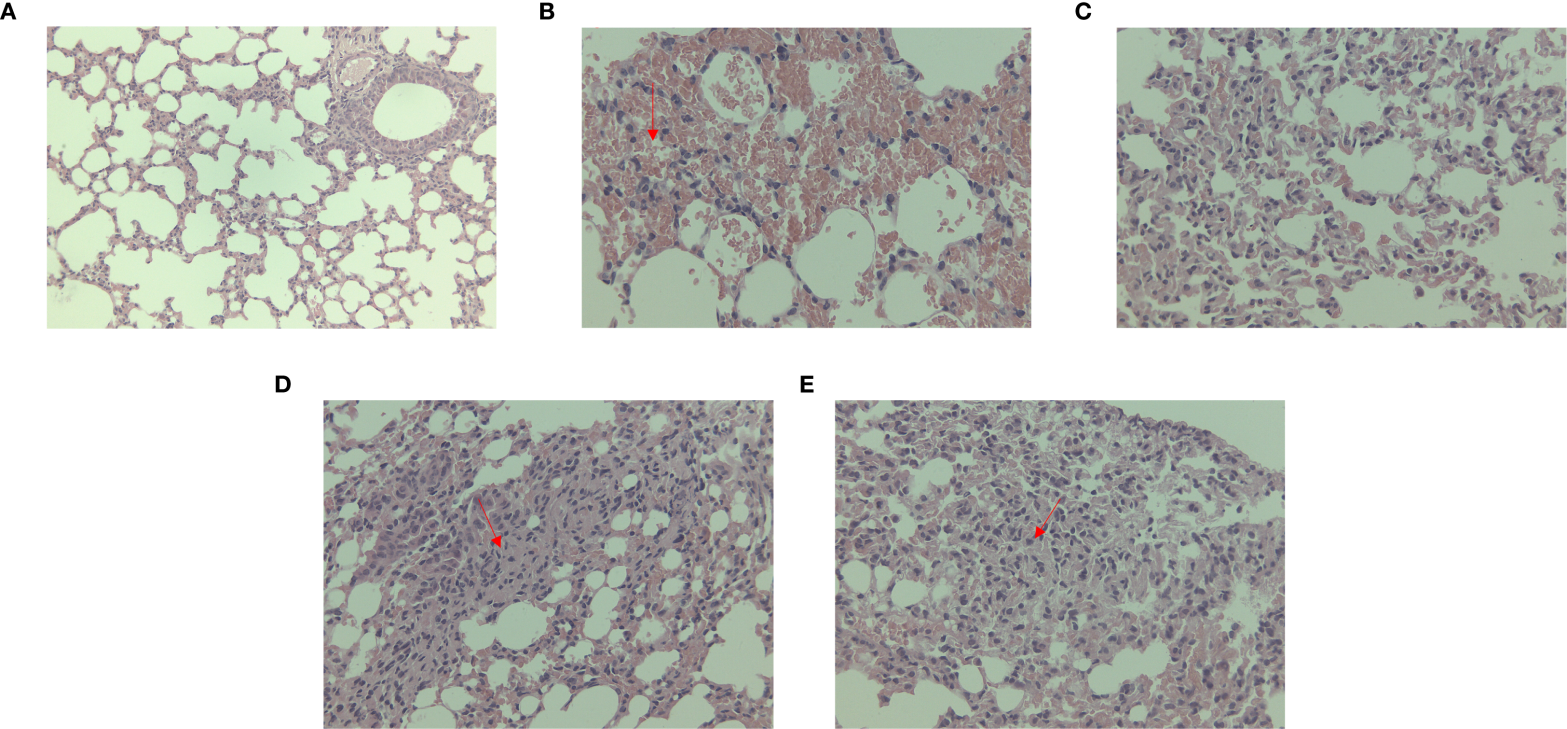
Lung lesions after coinfection with *Chlamydia psittaci* and *Enterococcus faecalis* in mice under microscopic observation. **(A)** Control group, **(B)** *C*. *psittaci* alone group, **(C)** *E. faecalis* alone group, **(D)** *C*. *psittaci*+*E. faecalis* group, and **(E)** *C*. *psittaci*/*E. faecalis* group.

Under microscopic observation, severe consolidation lesions were observed in the *C. psittaci*+*E. faecalis* group and *C. psittaci*/*E. faecalis* group, diffuse hemorrhagic inflammation was evident in the lungs of the *C. psittaci* alone group, and no obvious lesions were found in the *E. faecalis* alone group (**Figure 8**).

**Figure 9:**
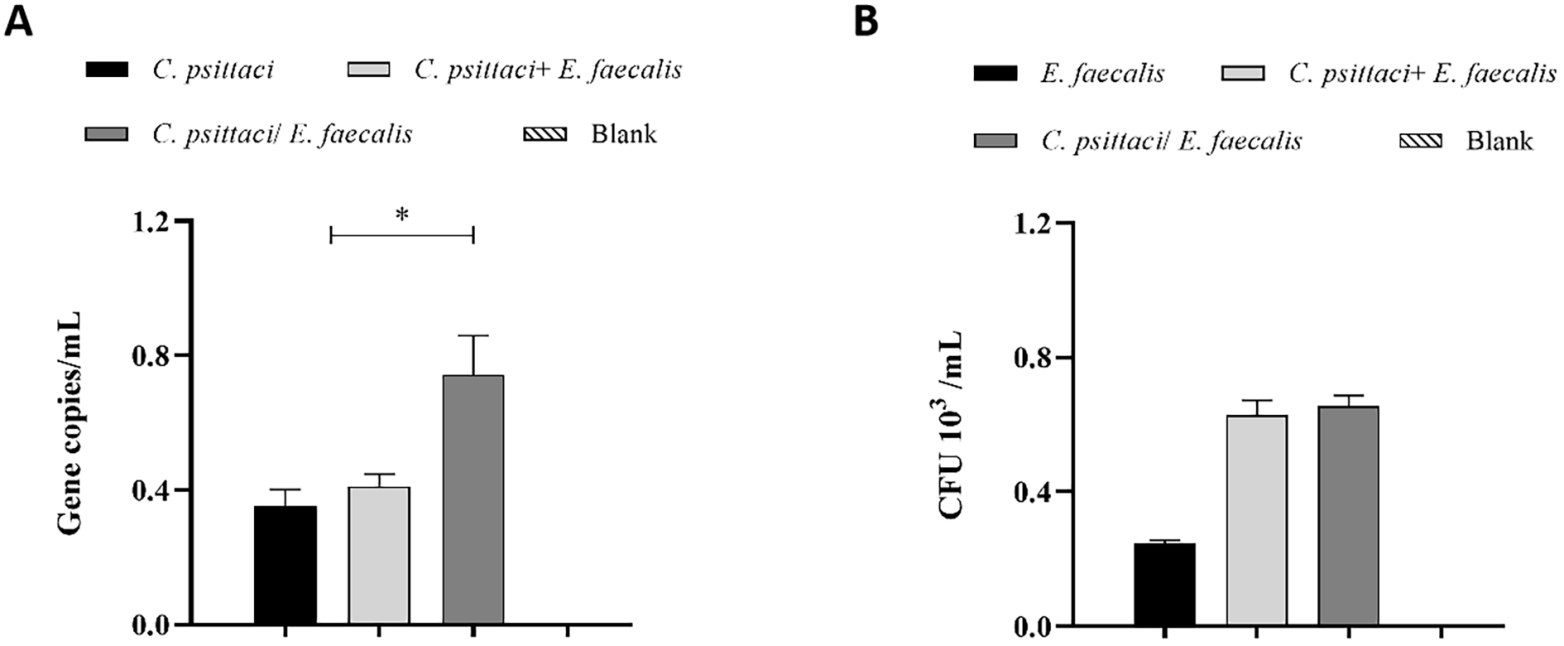
Determination of bacterial loads in the mouse lungs after coinfection with *Chlamydia psittaci* or *Enterococcus faecalis*. **(A)** *C*. *psittaci* loads in lungs on day 14 post-challenge. The symbol * indicates a statistically significant difference (p < 0.05). **(B)** *E. faecalis* loads in lungs on day 14 post-challenge. No significant difference in *E. faecalis* clearance was found between the *C*. *psittaci*/*E. faecalis* group and the *C*. *psittaci*+*E. faecalis* group **(B)**. The differences were analyzed using ANOVA (*p* < 0.05, *p* < 0.01).

Regarding bacterial loads, high chlamydial loads were determined in the *C. psittaci*/*E. faecalis* group compared to the *C. psittaci*+*E. faecalis* group or *C. psittaci* alone group (*p* < 0.05) (**Figure 9A**). On the contrary, no significant difference in *E. faecalis* clearance was found between the *C. psittaci*/*E. faecalis* group and the *C. psittaci*+*E. faecalis* group (**Figure 9b**).

## 4 Discussion

In the present study, positive genomics, *C. psittaci* isolate, and seropositive sera were determined in the patient’s samples, and *E. faecalis* was also recovered from his alveolar lavage and throat swabs. After therapy with doxycycline for 11 days, both high fever and respiratory distress were alleviated gradually, and the patient survived before the COVID-19 outbreak and quarantine in Wuhan. In the evidence-based animal study, both *C. psittaci*+*E. faecalis* and *C. psittaci*/*E. faecalis* infections aggravated mouse respiratory distress, characterized as diffuse hemorrhage, exudation, and fibrinous consolidation in mouse lungs, which were consistent with the white-like lungs of the patient after hospital admission. Therefore, primary infection with *C. psittaci* and secondary infection with *E. faecalis* may be associated with the patient’s respiratory distress.

Naturally, human *C. psittaci* infection is a typical zoonosis transmitted via inhalation of aerosolized particles from infected birds (Wang et al., 2024). The clinical manifestations range from asymptomatic illness to life-threatening pneumonia. *E. faecalis*, as a common commensal bacterium in the human gastrointestinal tract, is an opportunistic pathogen. It most often causes endogenous infections, such as urinary tract infections and bacteremia, when host immunity is compromised. One report implied that *E. faecalis* could cause pneumonia and lung abscesses in immunocompromised individuals (Mendes et al., 2020). However, coinfection of *C. psittaci* and *E. faecalis* remains elusive.

In our study, both *C. psittaci* and *E. faecalis* were identified in the patient with high fever. However, the definitive contribution of each pathogen to the severe pneumonia remained elusive. To our knowledge, human psittacosis can manifest as an asymptomatic syndrome with silent infection. Some employees from duck meat plants were reported to present flu-like symptoms or pneumonia after *C. psittaci* infection and recovered without treatment (Zhang et al., 2022). Severe symptoms in the patient may be associated with immunosuppression due to the presence of heart disease and type 2 diabetes for up to 7 years. The simultaneous onset of type 2 diabetes mellitus and heart disease significantly reduces the body’s immune response (Yap et al., 2019). The patient’s chronic disease may have contributed to his two-time high fever in the hospital. Recent analysis showed that immunocompromised patients overall had a 44% higher risk of death in hospital than patients with normal immune systems over the course of the COVID-19 pandemic. However, highly pathogenic *C. psittaci* infection could induce an immunocompromised state and aggravate H9N2 infection (Chu et al., 2016). In this study, the patient suffered from *C. psittaci*-elicited immunosuppression and chronic disease with low immunity and was sensitive to viral or bacterial infection. In the mouse model, *C. psittaci* infection caused hemorrhagic inflammation in the lungs, and a primary infection with *C. psittaci* followed by *E. faecalis* inoculation not only caused mice to develop more severe breathing difficulties than *C. psittaci* infection alone, but also caused a high number of lesions in the lungs of the *C. psittaci*/*E. faecalis* group. The above facts confirm our hypothesis that a primary infection with *C. psittaci* establishes a compromised lung environment that is severely exacerbated by secondary *E. faecalis* inoculation.

The potential role of *E. faecalis* is unclear over the patient’s disease course. Enterococci are rarely considered pulmonary pathogens, and they are considered colonizers of the airway. One patient was diagnosed with *E. faecalis*-associated acute primary lung abscess, highlighting *E. faecalis* as an etiologic agent in cases of non-resolving or complicated cases of pneumonia (Mendes et al., 2020). Moreover, epidemiological investigation confirmed a correlation between *E. faecalis* clones isolated from food-producing animals and human urinary tract infections caused by *E. faecalis* in France in 2016 (Abat et al., 2016). In slaughter animals, *E. faecalis* from human milk is claimed to be avirulent and antibiotic sensitive and does not breach the gut barrier (Anjum et al., 2022). However, some reports showed that *E. faecalis* harboring antibiotic resistance genes may contribute to bovine mastitis and act as a reservoir for the transmission of virulence factors to humans (Kim et al., 2022). In the present study, the *E. faecalis* isolate from the patient and his son may have originated from backyard chickens due to daily feeding activities. After inoculation into mice, hemorrhagic inflammation of the lungs was evident in both the *C. psittaci*+*E. faecalis* group and *C. psittaci*/*E. faecalis* group, but no typical lesion was found in the lungs infected with *E. faecalis* alone. Our animal model provided strong evidence that *E. faecalis*, which originated from animal-based food, may be an opportunistic human pathogen that worsens *C. psittaci* injury by increasing the burden of *C. psittaci* in the lungs. Moreover, it may be a real zoonotic pathogen with a potentially highly significant impact on human health.

The initial clinical and radiological presentation of the patient, characterized by high fever, bilateral ground-glass opacities, and hemorrhagic lesions, posed a significant diagnostic challenge, as it closely mimicked severe COVID-19 pneumonia and avian influenza (Zhao et al., 2021). This overlap in features, including the progression to “white lung” in severe cases (Coronaviridae Study Group of the International Committee on Taxonomy of Viruses, 2020; Attaway et al., 2021), underscores the critical importance of differential diagnosis in the context of a pandemic. Notably, the diffuse hemorrhagic inflammation observed in our coinfection mouse model differed from the typical fibrotic consolidation seen in advanced COVID-19 (Yin and Wunderink, 2018), suggesting distinct pathogenic mechanisms. In view of the rapid identification of human psittacosis from community-acquired pneumonia, RT-PCR and metagenomic next-generation sequencing are recommended for clinical diagnosis.

In conclusion, positive *C. psittaci* genomics and seroprevalence were identified in the patient. Subsequently, both *C. psittaci* and *E. faecalis* were isolated from the patient’s samples. After treatment with doxycycline, the patient recovered and was discharged from the hospital, suggesting that *C. psittaci* infection may have aggravated respiratory distress by aggravating his weak immunity. Our experimental data further demonstrate that secondary infection with *E. faecalis* can exacerbate respiratory distress and produce pathological lesions reminiscent of COVID-19. Various strategies must be urgently implemented to highlight this public health threat, in particular through the development and implementation of large surveillance systems based on animal and human health data to enable us to detect the prevalence of *C. psittaci* and *E. faecalis*.

## Data Availability

The datasets presented in this study can be found in online repositories. The names of the repository/repositories and accession number(s) can be found in the article/**Supplementary Material**.
