# Supplementary material for: Coinfection of Chlamydia psittaci and Enterococcus faecalis exacerbated respiratory distress in patients: from isolation to mouse model
Source: Front Cell Infect Microbiol. 2025 Oct 10;15:1662902. doi: 10.3389/fcimb.2025.1662902 (PMC12549663; doi:10.3389/fcimb.2025.1662902)

**Supplementary Table S1**

**Samples collected from the patient**

| **Sample ID** | **Sample types** | **Number** | **Date** |
| --- | --- | --- | --- |
| A-1 | Throat swab | 1 | 2019.12.25 |
| A-2 | Throat swab | 1 | 2019.12.25 |
| B-1 | Alveolar wash | 1 | 2019.12.13 |
| B-2 | Alveolar wash | 1 | 2019.12.13 |
| C | Tracheal mucosa | 1 | 2019.12.13 |
| D-1 | Sera | 1 | 2019.12.15 |
| D-2 | Sera | 1 | 2019.12.17 |
| D-3 | Sera | 1 | 2019.12.19 |
| D-4 | Sera | 1 | 2019.12.20 |
| D-5 | Sera | 1 | 2019.12.23 |
| Total | | 10 |  |

**Supplemental Table S2**

**Detecting pathogens from the patient and his closely contacts**

| **No.** | **Personal information** | **Gender** | **Ages** | **Samples** | **Clinical signs** |
| --- | --- | --- | --- | --- | --- |
| 1 | Duan (patient) | Male | 66 | Throat swab; alveolar wash; alveolar wash; Sera | Prolonged high fever; dry coughing with asthma, dizziness, headache, nausea, dyspnea and chest pains |
| 2 | Spouse | Female | 63 | Throat swab; sera | Healthy |
| 3 | Son | Male | 35 | Throat swab; sera | Prolonged high fever; dry coughing; headache |
| 4 | Older granddaughter | Female | 9 | Throat swab; sera | Healthy |
| 5 | Little granddaughter | Female | 6 | Throat swab; sera | Healthy |
| 6 | Daughter in law | Female | 35 | Throat swab; sera | Healthy |
| 7 | Nurse | Female | 27 | Throat swab; sera | Healthy |
| 8 | Doctor | Male | 29 | Throat swab; sera | Healthy |

**Supplementary Table S3**

**Primers of sequences used for detecting COVID-19**

| **S/N** | **Target gene** | **Oligonucleotide ID** | **Sequence (5'-3')** | **Specific marks** |
| --- | --- | --- | --- | --- |
| 1 | *RdRP gene* | RdRP_SARSr-F2 | GTGARATGGTCATGTGTGGCGG | 600 nM per reaction |
|  |  | RdRP_SARSr-R1 | CARATGTTAAASACACTATTAGCATA | 800 nM per reaction |
|  |  | RdRP_SARSr-P2 | FAM-CAGGTGGAACCTCATCAGGAGATGC- BBQ | Specific for 2019-nCoV, 100 nM per reaction and mix with P1 |
|  |  | RdRP_SARSr-P1 | FAM- CCAGGTGGWACRTCATCMGGTGATGC- BBQ | Pan Sarbeco-probe for 2019-nCoV, SARS-CoV, bat-SARS-related CoVs,100 nM per reaction and mix with P2 |
| 2 | *E gene* | E_Sarbeco_F1 | ACAGGTACGTTAATAGTTAATAGCGT | 400 nM per reaction |
|  |  | E_Sarbeco_F1 | ATATTGCAGCAGTACGCACACA | 400 nM per reaction |
|  |  | E_Sarbeco_P1 | ATATTGCAGCAGTACGCACACA | 200 nM per reaction |

**Supplemental Table S4**

**Animal experimental designs**

| **Groups** | **Numbers** | **Inoculated schedule** | **Dose** | **Route** |
| --- | --- | --- | --- | --- |
| 1 | 8 | *C. psittaci* alone | 1x10^5^ IFU/100 µL | i.n^a^ |
| 2 | 8 | *E. faecalis* alone | 1x10^7^CFU/100 µL | i.n |
| 3 | 8 | *C. psittaci + E. faecalis* simultaneously | 1x10^5^ IFU+1x10^7^ CFU /100 µL | i.n |
| 4 | 8 | *C. psittaci first, E. faecalis* 3 days later  (*C. psittaci*/ *E. faecalis*) | 1x10^5^ IFU/1x10^7^ CFU/100 µL | i.n |
| 5 | 8 | PBS control | 100 µL | i.n |

^a^ Intranasal administration

**Supplementary Figure S1**

**Body temperature curve during patient’s hospital admission.**

First high fever occurred from December 13 to December 20, 2019 after patient was treated with Ceftazidime and Caspofungin via vein injection. Afterwards, patients received Levofloxacin and Cefoperazone from December 18th to December 21 and the body temperature maintained normal level on December 24, 2019. After the second high fever resumed and patient was treated with Doxycycline from December 25, 2019 to January 6, 2020.


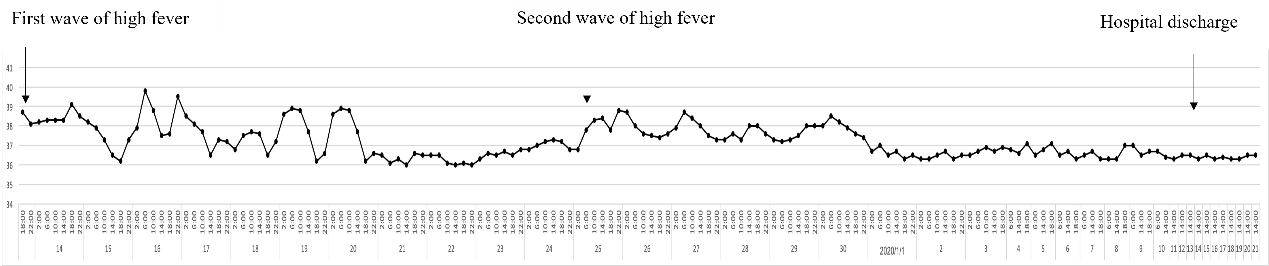


**Supplementary Figure S2**

**Chest X-ray for patient.**

(a) The patient displayed exudate inflammations, ground-glass opacity in two lungs and white lung-like lesions were evident upon hospital admission. (b) After therapy with Doxycycline via intravenous injection, ground-glass lesions were reduced. (c) Obvious pulmonary consolidation was observed on January 1, 2020.


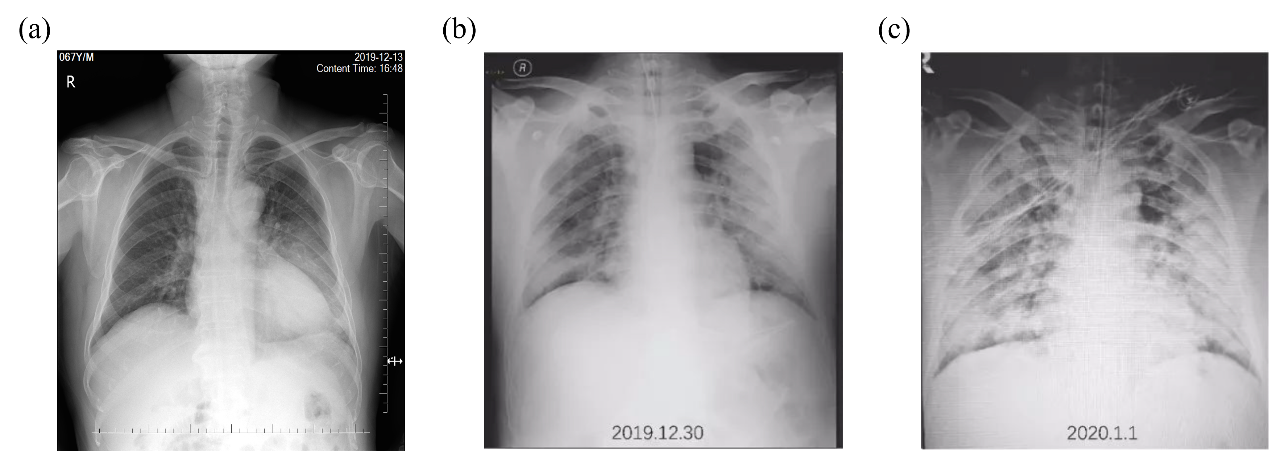

Supplement: Supplementary file 1 [file Table1.docx]
